# Impact of Impella on Coronary Flow Assessed by Transthoracic Doppler Echocardiography

**DOI:** 10.7759/cureus.46604

**Published:** 2023-10-06

**Authors:** Masahiro Hada, Eisuke Usui, Nobutaka Wakasa, Tetsuo Sasano, Tsunekazu Kakuta

**Affiliations:** 1 Department of Cardiovascular Medicine, Tsuchiura Kyodo General Hospital, Ibaraki, JPN; 2 Department of Cardiovascular Medicine, Tokyo Medical and Dental University, Tokyo, JPN

**Keywords:** coronary microcirculation, doppler echocardiography, coronary blood flow, myocardial infarction, impella device

## Abstract

A 66-year-old male patient presented with anterior ST-elevated myocardial infarction and cardiogenic shock. After placement of the Impella device (Abiomed, Danvers, Massachusetts), the patient successfully underwent percutaneous coronary intervention for lesions in the left anterior descending artery (LAD) and left circumflex artery. Coronary flow in the LAD according to the support setting was evaluated using transthoracic Doppler echocardiography during Impella weaning.

## Introduction

The Impella device is a percutaneous micro-axial continuous-flow pump that is used for patients with cardiogenic shock and left ventricular (LV) unloading [1]. Impella-assisted forward blood flow may increase coronary blood flow; however, only a few studies have investigated in vivo coronary flow with Impella support [2]. We present a case involving a patient with myocardial infarction and subsequent cardiogenic shock whose coronary blood flow in the left anterior descending artery (LAD), according to the support setting, was evaluated using transthoracic Doppler echocardiography during Impella weaning.

## Case presentation

**Figure 1:**
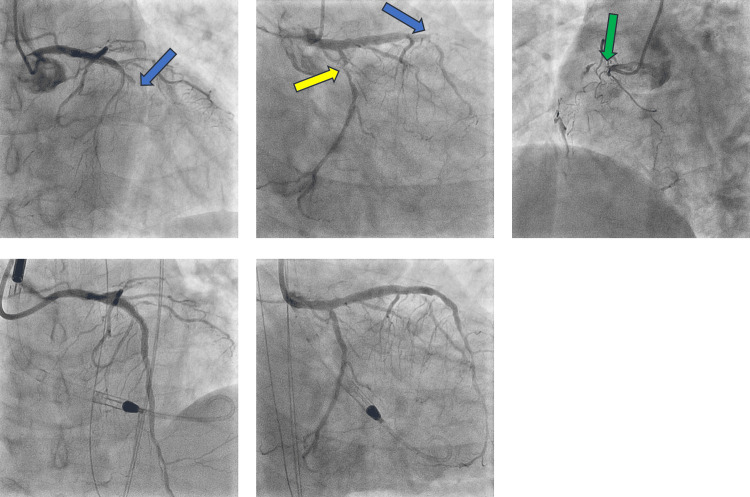
Coronary angiography and percutaneous coronary intervention Coronary angiography showed totally occlusive lesions in the LAD and RCA and another 90% stenosis in the proximal LCx. Ultimaster 3.5 × 33 mm was deployed in the LAD, and Combo 2.5 × 18 mm was implanted in the LCx with Impella support. The blue arrow shows total occlusion of LAD, the yellow arrow shows 90% stenosis in LCx, and the green arrow shows total occlusion of RCA. LAD: Left anterior descending artery, LCx: Left circumflex artery, RCA: Right coronary artery

A 66-year-old male patient with a history of hypertension and dyslipidemia presented to our emergency department with sudden-onset chest pain when sleeping. Electrocardiography showed ST segment elevation in II, III, aVF, and V2-6. Echocardiography revealed diffuse left ventricular wall hypokinesis (ejection fraction of 30%) with severe hypokinesis in the anterior wall. The patient was transferred to the catheterization laboratory for primary percutaneous coronary intervention (PCI) with a diagnosis of anterior ST-elevation myocardial infarction. At the commencement of angiography, the patient’s blood pressure was 71/42 mmHg under catecholamine use of 7γ of dobutamine and 0.25γ of noradrenalin. Also, he was administered additional bolus noradrenalin o.o2 mg intravenously. Coronary angiography revealed the presence of total occlusions in the mid-LAD and proximal right coronary arteries and another 90% stenosis in the proximal left circumflex artery (LCx) (Figure 1).

The patient exhibited signs of cardiogenic shock, and the Impella CP Smart Assist system (Abiomed, Danvers, Massachusetts) was placed through his right common femoral artery before the high-risk PCI. The PCI was performed for the culprit LAD and non-culprit LCx lesions. Under Impella support, a guidewire was crossed distal to the LAD lesion, and pre-dilatation was performed with a 2.5-mm semi-compliant balloon. A third-generation drug-eluting stent (DES) (Ultimaster Nagomi 3.5 × 33 mm, Terumo Interventional Systems, Tokyo, Japan) was deployed with intravascular ultrasound (IVUS) imaging guidance. Post-dilatation was performed with 3.25- and 4.0-mm non-compliant balloons for the mid-distal lesion and proximal segment, respectively. IVUS showed acceptable stent expansion and apposition. After pre-dilatation with a 2.5-mm semi-compliant balloon for the LCx lesion, a third-generation DES (Combo plus 2.5 × 18 mm, OrbusNeich Medical, Florida, USA) was implanted. Angiography revealed thrombolysis in myocardial infarction grade 3 flow in both the LAD and LCx.

After successful PCI for the LAD and LCx lesions, the coronary flow velocity of the LAD assessment was assessed using transthoracic Doppler echocardiography in the Impella weaning phase.

Investigations

Our study was approved by the institutional ethics committee (reference no. 2023FY42/Tsuchiura; June 5, 2023) and complied with the tenets of the Declaration of Helsinki for investigations involving humans. The patient provided written informed consent for this study and the use of the data in future analyses.

Echocardiographic studies were performed according to the American Society of Echocardiography guidelines using an ultrasound system (GE Vivid E95; GE Vingmed Ultrasound, Horten, Norway) with a multifrequency transducer and second-harmonic technology, as previously reported [3,4]. Briefly, after a standard examination, the coronary flow in the mid-distal portion of the LAD was visualized in a modified three-chamber view [5]. For color flow mapping, the velocity range was set at 16-24 cm/sec. A sample volume (3-5 mm wide) was selected at the mid to distal LAD to measure blood flow velocity. Basal diastolic peak coronary flow velocity (bDPV) was first measured under Impella support at P8 and subsequently at P4 and P2. After the Impella removal, the diastolic peak velocity (DPV) under maximal hyperemia (hDPV) induced by intravenous adenosine (140 μg/kg per min through a central vein) was measured. All data were digitally stored for offline data analysis. Three optimal flow signal profiles at rest and during hyperemia were obtained offline from the recorded data. Coronary flow velocity reserve (CFVR) was calculated as the hyperemic peak diastolic flow velocity ratio to the basal peak diastolic flow velocity using the software package of the ultrasound system [6]. Two Doppler echocardiography experts who were blinded to the clinical data separately analyzed all stored data at a one-week interval and performed the analyses twice to evaluate the reproducibility of the echocardiography-derived data.

**Figure 2:**
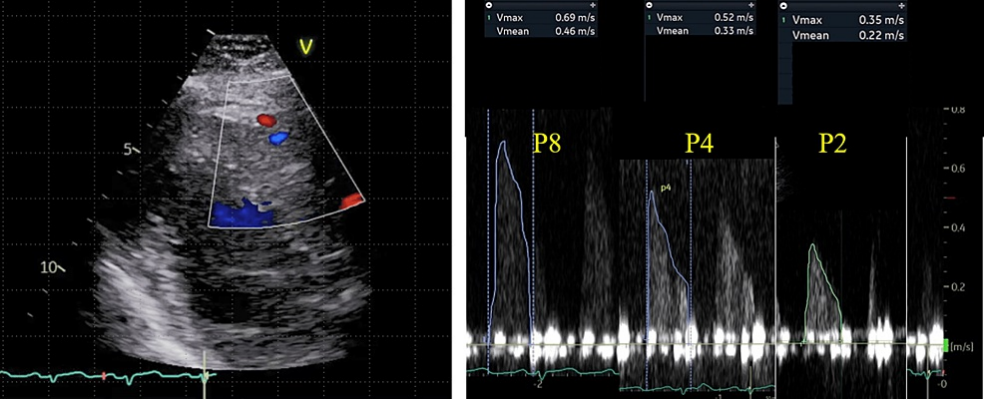
Coronary flow of the LAD during Impella support Coronary flow in the mid-distal portion of the LAD was assessed using a modified three-chamber view. A sample volume (3-5 mm wide) was positioned on the distal LAD to measure blood flow velocity. bDPV was 35 cm/s at P2, 52 cm/s at P4, and 69 cm/s at P8. The peak LAD flow velocity gradually decreased with the reduction in the Impella support level. LAD: left anterior descending artery, bDPV: basal Diastolic peak velocity

bDPV was 69 cm/s at P8 (3.7 l/min), 52 cm/s at P4 (2.7 l/min), and 35 cm/s at P2 (2.3 l/min) (Figure 2).

The peak velocity gradually decreased with the reduction in the Impella support level. However, systemic blood pressure and heart rate remained stable and showed no significant change, indicating 109/70 mmHg and 101/min at P8, 114/70 mmHg and 97/min at P4, and 104/63 mmHg and 98/min at P2.

**Figure 3:**
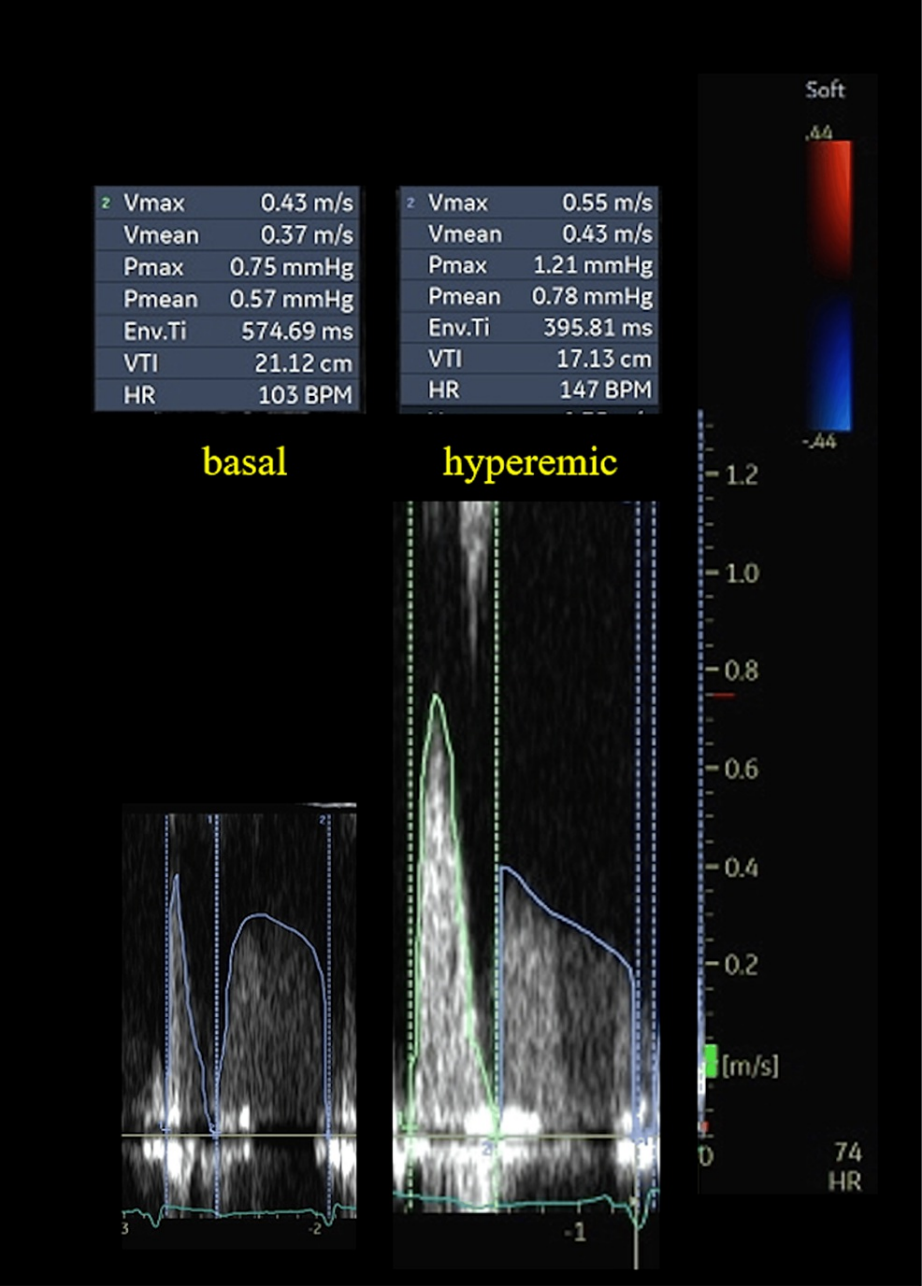
Basal and hyperemic coronary flow after the Impella removal After the Impella removal, basal and hyperemic DPV of the LAD were assessed. The bDPV and hDPV were 43 and 55 cm/s, respectively, and the CFVR was 1.28. LAD: left anterior descending artery, bDPV: basal diastolic peak velocity, hDPV: hyperemic diastolic peak velocity

On day 13, bDPV and hDPV after the Impella removal were assessed. The bDPV and hDPV were 43 and 55 cm/s, respectively. The CFVR was 1.28 (Figure 3).

Management

On day one, peak creatin kinase was 11669 U/L at 3 hours after primary PCI. The Impella was removed on day two, considering the patient’s hemodynamic stability, and extubation was performed on day three. The patient was treated with guideline-directed medical therapy, including double antiplatelet, statin, angiotensin-converting enzyme inhibitor, diuretics, and a low-dose β blocker. Cardiac rehabilitation was initiated, and the patient showed a good clinical course without adverse events. Finally, the patient was discharged on day 14.

## Discussion

The Impella device is a percutaneous transvalvular microaxial flow pump currently used for cardiogenic shock and high-risk PCI to facilitate left ventricular unloading [1,7]. It provides superior hemodynamic support compared to intra-aortic balloon pumping [8,9]. However, the coronary flow during Impella support and the change in coronary flow according to the Impella support level are not well elucidated. Alrarqaz et al. studied the coronary pressure with a pressure wire before high-risk PCI with mechanical circulatory support using Impella. They concluded that a significant increase in distal coronary pressure beyond the lesions and a decrease in left ventricular end-diastolic pressure were observed with maximum Impella support [10]. Another report assessed the coronary pressure and velocity of non-stenotic vessels using intracoronary wire after Impella-supported PCI. The authors reported that the pressure index of FFR remained unchanged when the support level of Impella increased. On the other hand, they observed that hyperemic velocity increased and microvascular resistance decreased. By unloading LV, the coronary pressure increase and microvascular resistance decrease may occur simultaneously [11].

This is the first report that describes the coronary flow velocity using transthoracic Doppler echocardiography in a patient with myocardial infarction-induced cardiogenic shock during Impella support and its weaning phases, as well as after Impella removal.

## Conclusions

This case demonstrated that the Impella positively affected coronary microcirculation following a change in support flow without significant alterations in systemic blood pressure. LV unloading by the Impella support may decrease LV end-diastolic pressure and reduce sub-endocardial pressure, which might increase the epicardial coronary flow observed in this case without significant changes in the systemic blood pressure and heart rate according to the reduction of microcirculatory resistance. Our case illustrates how the Impella can influence LV unloading with the reduction of LV myocardial oxygen demand but also with an increase in oxygen supply through enhanced coronary flow, primarily by impacting coronary microcirculation.
